# Perfusion of isolated rat kidney with Mesenchymal Stromal Cells/Extracellular Vesicles prevents ischaemic injury

**DOI:** 10.1111/jcmm.13249

**Published:** 2017-06-21

**Authors:** Marilena Gregorini, Valeria Corradetti, Eleonora Francesca Pattonieri, Chiara Rocca, Samantha Milanesi, Andrea Peloso, Silvana Canevari, Loris De Cecco, Matteo Dugo, Maria Antonietta Avanzini, Melissa Mantelli, Marcello Maestri, Pasquale Esposito, Stefania Bruno, Carmelo Libetta, Antonio Dal Canton, Teresa Rampino

**Affiliations:** ^1^ Unit of Nephrology Dialysis and Transplantation Fondazione IRCCS Policlinico San Matteo Pavia Italy; ^2^ Department of Internal Medicine and Therapeutics University of Pavia Pavia Italy; ^3^ PhD School of Experimental Medicine University of Pavia Pavia Italy; ^4^ Unit of General Surgery Fondazione IRCCS Policlinico San Matteo Pavia Italy; ^5^ Department of Experimental Oncology and Molecular Medicine Fondazione IRCCS Istituto Nazionale dei Tumori Milan Italy; ^6^ Cell Factory and Research Laboratory‐Department of Pediatrics Fondazione IRCCS Policlinico San Matteo Pavia Italy; ^7^ Department of Molecular Biotechnology and Health Sciences University of Torino Torino Italy

**Keywords:** ischaemic injury, kidney perfusion, stem cells, extracellular vesicles, microarray analysis

## Abstract

Kidney donation after circulatory death (DCD) is a less than ideal option to meet organ shortages. Hypothermic machine perfusion (HMP) with Belzer solution (BS) improves the viability of DCD kidneys, although the graft clinical course remains critical. Mesenchymal stromal cells (MSC) promote tissue repair by releasing extracellular vesicles (EV). We evaluated whether delivering MSC‐/MSC‐derived EV during HMP protects rat DCD kidneys from ischaemic injury and investigated the underlying pathogenic mechanisms. Warm ischaemic isolated kidneys were cold‐perfused (4 hrs) with BS, BS supplemented with MSC or EV. Renal damage was evaluated by histology and renal gene expression by microarray analysis, RT‐PCR. Malondialdehyde, lactate, LDH, glucose and pyruvate were measured in the effluent fluid. MSC‐/EV‐treated kidneys showed significantly less global ischaemic damage. In the MSC/EV groups, there was up‐regulation of three genes encoding enzymes known to improve cell energy metabolism and three genes encoding proteins involved in ion membrane transport. In the effluent fluid, lactate, LDH, MDA and glucose were significantly lower and pyruvate higher in MSC/EV kidneys as compared with BS, suggesting the larger use of energy substrates by MSC/EV kidneys. The addition of MSC/EV to BS during HMP protects the kidney from ischaemic injury by preserving the enzymatic machinery essential for cell viability and protects the kidney from reperfusion damage.

## Introduction

The pool of kidneys currently available for transplantation could be expanded with the procurement of organs from the DCD. Nevertheless, the use of DCD kidneys is limited by a high rate of primary non function and delayed graft function and acute rejection, which results in a worse clinical course 1, 2, 3, 4, 5, 6, 7, 8. Ischaemia occurring in DCD kidneys causes metabolism slowdown and the consumption of ATP and adenosine diphosphate residual pools. The injury caused by ischaemia is worsened during reperfusion due to the accumulation of free radicals and reactive oxygen species (ROS) 9, 10, 11, 12, 13, 14. HMP with BS improves DCD kidney viability by providing metabolic sources for ATP generation and glutathione, which protect against ROS 15, 16, 17; nevertheless, the graft clinical course remains poor 3, 4, 5. MSC are multipotent cells that abate immune and inflammatory responses and promote tissue repair also by releasing EV 18, 19, 20, 21, 22, 23, 24, 25, 26, 27, 28, 29. We have already shown that MSC injection in a renal transplant model protects the graft by reducing ischaemia/reperfusion injury and acute rejection and guides the cytokine network towards a tolerogenic one 30, 31, 32, 33. The originality of our study resides in a new application of MSC, consisting in pre‐transplant graft perfusion. The core rational behind this application is that delivering MSC‐ or MSC‐derived EV (hereafter named EV) to the isolated kidney, as part of the HMP procedure, prepares renal cells and the environment to face the incoming injury, rather than contrasting the assault once it has fired up. Thus, we investigated this novel approach in a rat DCD model and evaluated the morphological, biochemical and molecular effects of MSC/EV on perfused kidneys.

## Materials and methods

### Animals

Fisher F344 (F) rats were used as kidney donors (Charles River, Lecco, Italy). Transgenic Sprague Dawley (SD) rats expressing Enhanced Green Fluorescence Protein (EGFP) 34 (Japan Slc, Hamamatsu, Japan) were used as MSC donors. Rats weighed between 125 and 150 g. Animals were handled according to the guidelines of the Italian Health Ministry (n° 339/2016‐PR).

### MSC expansion and characterization

MSC were isolated from bone marrow of EGFP transgenic SD rats, expanded *in vitro* and used at P2/P3 as previously described 35.

EGFP‐rat MSC, hereafter referred to as MSC, were characterized for plastic adhesion, morphology, antigen surface expression of CD49e, CD90 and CD29 and the absence of CD45 and CD11b (all antibodies were purchased from BioLegend, San Diego, CA, USA) performed with a Navios flow cytometer (Beckman Coulter, Milan, Italy) and differentiation capacity 35.

### EV isolation and characterization

EV were obtained from supernatants of MSC at 80% confluence, as previously described 26. Briefly, MSC were cultured overnight in D‐MEM (Gibco, Life Technologies, Milan, Italy) without foetal calf serum (FCS). Supernatants were centrifuged at 3,000 × g for 20 min. to remove cellular debris, and cell‐free supernatants were then centrifuged twice at 100,000 × g for 1 hr at 4°C.

Fluorescent beads ranging in size from 0.1 to 1 μm (Megamix; BioCytex, Marseille, France) were employed to precisely gate EV. As EV derived from MSC express surface molecules that are characteristic of the cells of origin, anti‐rat CD49e (as positive marker) and anti‐rat CD45 (as negative marker) (both from BioLegend) were used. The analysis was performed by direct immunofluorescence with a Navios flow cytometer (Beckman Coulter), and the data were analysed using Kaluza software. Moreover, some specific exosomal markers, such as CD63, CD9 and CD81 (Miltenyi Biotec, Bergisch Gladbach, Germany), were also analysed, using the Guava easyCyte FlowCytometer (Millipore, Billerica, MA, USA) with InCyte software.

### MSC viability

To test whether hypothermia affects MSC activity, after exposition at 4°C for 2 and 4 hrs, cell viability was evaluated with the Trypan blue exclusion test. Viable cells had a clear cytoplasm, whereas non‐viable cells had a blue cytoplasm. The viability percentage was calculated = [number of viable cells/ total n. of cells (viable + non‐viable)] × 100.

### *In vivo* experiments

Using the rat DCD kidney model, rats were anaesthetized using Isoflurane 2–5% (Baxter, Como, Italy). After a midline laparotomy, the left retroperitoneal renal area was exposed and the lumbar arteries were isolated and sectioned; subsequently, the renal artery and vein were isolated. After 20 min. of warm ischaemia obtained by renal artery clamping, the left nephrectomy was completed with the preservation of the renal hilum. Kidneys were then perfused with BS (*n* = 5), or with BS supplemented with 3 million MSC (*n* = 5), or BS supplemented with EV isolated from 3 million MSC (*n* = 5). Continuous perfusion was performed for 4 hrs at 4°C, and then, the effluent fluid was collected and stored at −20°C. Kidneys were split into two aliquots, one fixed in 10% formalin for morphological studies and the other frozen in liquid nitrogen for RT‐PCR. For the microarray analysis, we also studied another group of non‐perfused kidneys (*n* = 5) (NP) obtained after 20 min. of warm ischaemia and preserved in RNA later (Ambion, Austin, TX, USA).

### Renal histopathology

#### EGFP expression

To track MSC, EGFP renal expression was studied by immunohistochemistry as already described 35. Briefly, 3‐μm‐thick sections of paraffin embedded tissue were collected on poly‐L‐lysine‐coated slides (Dako, Glostrup, Denmark). These were dewaxed in xylol, passed in a decreasing series of alcohol and rehydrated with distilled water. Endogenous peroxidase was blocked with H_2_O_2_/methanol 3.7% vol/vol for 10 min. followed by H_2_O_2_. After three washings in PBS, the sections underwent microwave antigen retrieval, then were exposed overnight at 4°C to monoclonal mouse anti‐green fluorescent protein antibody IgG1 (Chemicon International, Billerica, MA, USA). After three washings in PBS, the immunocomplex was visualized with the biotin–streptavidin–peroxidase complex and 3,3‐diaminobenzidine (Dako). Sections were counterstained with Harris haematoxylin. Negative controls included both omission of the primary Ab and substitution of IgG for primary antibodies. Kidney sections of SD‐EGFP were used as positive controls. We counted EGFP‐positive cells/HPF (×400) in 10 renal sections per kidney.

### Renal morphology

Twenty subserial cross‐sections of each kidney were stained with periodic acid–Schiff (PAS) and examined by two investigators in a double‐blind fashion, using an Olympus IX8 microscope connected to a CCD camera and the software imaging analysis Cell‐R.

Renal damage was evaluated by counting the percentage of tubules/HPF in at least 10 non‐consecutive fields presenting with the following lesions: tubular epithelial cell flattening, brush border loss (BBL), bleb formation (BF), tubular necrosis (TN) and tubular lumen obstruction (TO) 36. Tubular epithelial cell flattening (TF) and BBL were classified as mild lesions; BF, TN and TO as severe lesions.

The global renal damage score, as described by Paller *et al*. 12, was obtained by assigning each lesion a different score: TF(1 point), BBL (1 point), cell membrane BF (2 points), TN (2 points) and TO (2 points). When two or more lesions were present in the same tubule, the most severe score was assigned.

### Biochemical assays

In effluent fluid, malondialdehyde (MDA) amounts were quantified with the HPLC method using the Chromosystems assay kit (Chromosystems GmbH, Gräfelfing, Germany). Glucose, lactate and LDH were quantified with a Clinical Chemistry Analyser (ARCHITECT, Abbot, Italy), and the pyruvate concentration was measured by spectrophotometry (Beckman Coulter). All experiments were quadruplicated.

### RNA extraction and RT‐PCR in BS, MSC/EV kidneys

Total RNA was extracted using the TRIzol method. RNA was treated with DNase from the RNase‐Free DNase Set (Qiagen, Hilden, Germany) and dissolved in nuclease free water. Extracted RNA was tested for quantity and integrity by spectrophotometric analysis (NanoDrop; Thermo Scientific, Waltham, MA, USA). A total of 1 μg of RNA per condition was reverse transcribed into cDNA with the 1st Strand cDNA Synthesis Kit for RT‐PCR (AMV) (Roche Applied Science, Penzberg, Germany). cDNA was used to perform the real‐time PCR analyses in 96‐well optical reaction plates, using ABI prism 5700 (Applied Biosystems, Waltham, MA, USA) and the 5‐exonuclease assay (TaqMan technology) in a 10 μl reaction volume containing TaqMan Universal Master Mix, optimized concentrations of FAM‐labelled probes for B2 m, Idh2, Ndufs8, Pdhb, Calb1, Slc16a1 and Atp6v0d2 (Applied Biosystems, catalogue numbers Hs00187842_m1, Hs00158033_m1, Hs00159597_m1, Hs00168650_m1, Hs01077197_m1, Hs01560299_m1 and Hs00403032_m1, respectively). Water replacing the cDNA was included in the real‐time PCR as a control. The results were analysed using a comparative method, and values normalized to the β2 m expression and converted into fold change. All experiments were quadruplicated.

### Microarray analysis

To infer the transcriptional changes induced by perfusion, in a preliminary experiment, after warm ischaemia, five non‐perfused kidneys (NP) and five MSC‐perfused kidneys (MSC) were stored in RNA later and processed for microarray gene expression analysis.

RNA was extracted using the RNeasy Mini Kit (Qiagen) according to the manufacturer's instructions. RNA quality was assessed using Bioanalyser (Agilent, Santa Clara, CA, USA) and was quantified using spectrophotometry (Nanodrop, DE). Total RNA was amplified using the Agilent Low Input Quick Amp WT labelling kit according to the manufacturer's instructions. Briefly, 100 ng of total RNA was used to synthesize double‐stranded cDNA, which was amplified by *in vitro* transcription and labelled with cy3‐dCTP. Fluorescent dye‐labelled cRNA was hybridized to SurePrint G3 Rat GE 8 × 60K Microarray, containing probes recognizing about 30,000 transcripts designed on RefSeq Build 36.2. Hybridization and washing were performed on the Agilent's Microarray Platform according to Agilent's standard protocols. Microarray images were acquired using the Agilent DNA microarray scanner. Raw gene expression data were generated using the Agilent feature extraction software and were preprocessed using the *limma* package 37. Briefly, raw data were log2 transformed and normalized using the function *normalize Between Arrays* with cyclic loss normalization. Normalized data were filtered according to the following procedure: for each array, probes were referred to as ‘expressed’ if they had an intensity signal greater than 10% of the 95th percentile of the negative control probes, then we filtered out probes referred to as ‘expressed’ in less than four samples. After filtering, replicated probes were summarized by calculating their average expression. Finally, we collapsed genes targeted by multiple probes using the ‘maxRowVariance’ method implemented in the collapse Rows function of the WGCNA package 38.

Genes differentially expressed between biological classes were identified using the linear model approach with the empirical Bayes method implemented in the *limma* package. Multiple‐testing correction was performed using the Benjamini–Hochberg false discovery rate (FDR). Genes with an FDR < 0.05 were considered significant.

To identify known biological pathways altered between biological classes, we ran a gene set enrichment analysis (GSEA) using GSEA v2.0.13 software 39. Gene sets retrieved from MSigDB (canonical pathways, C2.cp.v5.1 collection) were tested for enrichment, and those with a *P*‐value <0.001 and an FDR < 0.05 were selected as significantly enriched. GSEA results were visualized using Cytoscape v2.8.3 software and the Enrichment Map plugin. Core genes were identified by leading edge analysis of significantly enriched gene sets. All microarray data were MIAME compliant and were deposited into the NCBI's GEO database (http://www.ncbi.nmlm.nih.gov/projects/geo/) with accession number GSE84563.

### Statistical analysis

GraphPad Prism software (San Diego, CA, USA) was used for the statistical analyses. Biochemical measures were compared using the Student's unpaired *t*‐test. anova followed by the Newman–Keuls test was applied to compare continuous variables among more than two groups. A *P* < 0.05 was considered statistically significant.

## Results

### Rat MSC and EV characterization

**Figure 1:**
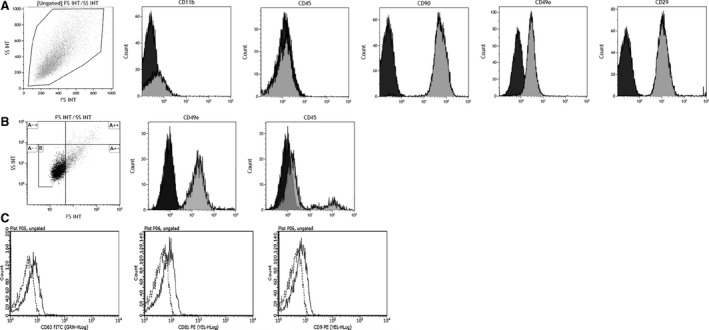
Rat MSC and EV cytofluorimetric characterization. Panel **A**. Immunophenotype of one representative expanded rat EGFP‐MSC culture. Representative dot plot of MSC gated by physical parameters and histograms of surface marker expression (grey ispots) and isotypic controls (black ispots). Rat MSC are negative for CD11b and CD45 and positive for CD90, CD49e and CD29. Panel **B**. Representative dot plot of EV gated by dimension parameters, using 1‐μm calibration beads. Representative histograms of surface marker expression (grey ispots) and isotypic controls (black ispots). Rat EV were positive for CD49e and negative for CD45. Panel **C**. Representative histograms of specific rat EV markers (continuous line) and isotypic controls (broken line). Rat EV were positive for CD63, CD81 and CD9.

MSC showed the typical spindle shape morphology and maintained their capacity to *in vitro* differentiate into osteoblasts and adipocytes as confirmed by the presence of mineralization nodules and fat droplets with histological staining, as already described 35. MSC resulted positive for CD49e, CD90 and CD29, while they were negative for CD45 and CD11b (Fig. 1A). EV were CD49e positive and CD45 negative (Fig. 1B), and expressed some specific exosomal markers, such as CD63, CD9 and CD81 (Fig. 1C).

Short‐term hypothermia marginally affected MSC viability; in fact, the percentage of viable MSC after exposure at 4°C for 2 and 4 hrs was 86% and 84%, respectively.

### EGFP‐MSC homing tracking

**Figure 2:**
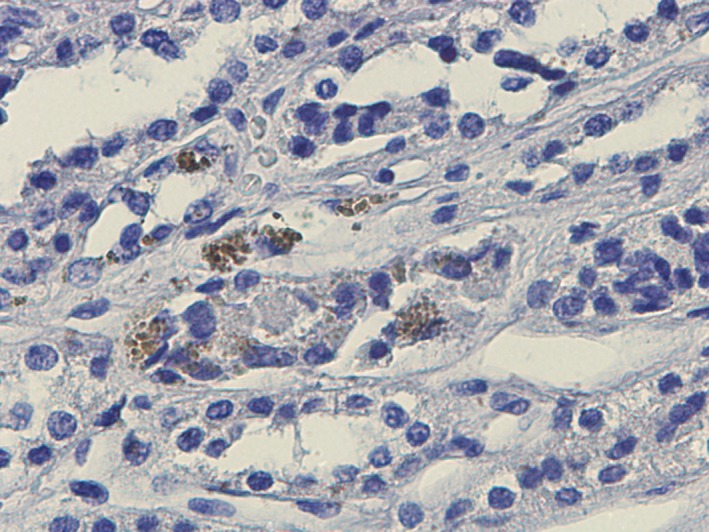
Homing tracking of rat EGFP‐MSC. Representative renal section of MSC‐perfused kidneys. EGFP immunohistochemistry showing tubular and vascular localization of MSC. Magnification ×400.

In MSC‐perfused kidneys, EGFP staining (5–10 cells per section) was tracked in vessels, tubules and interstitium (Fig. 2). There was no evidence of macro/microvascular engorgement or thrombosis.

### Renal ischaemia damage was more severe in kidneys perfused with BS than MSC or EV

**Figure 3:**
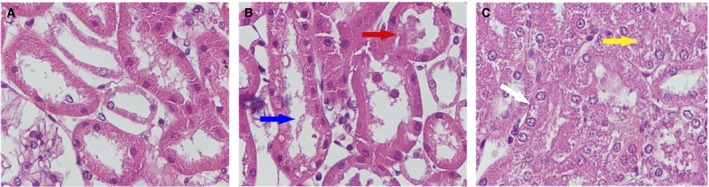
Renal lesions evaluated in the DCD model. Formalin‐fixed tissue stained with PAS. Panel **A**. Tubular epithelial cell flattening (TF). Panel **B**. Blue arrow indicates brush border loss (BBL), and red arrow indicates bleb formation (BF). Panel **C**. Yellow arrow indicates tubular lumen obstruction (TO) and white arrow tubular necrosis (TN). Magnification ×400.

**Figure 4:**
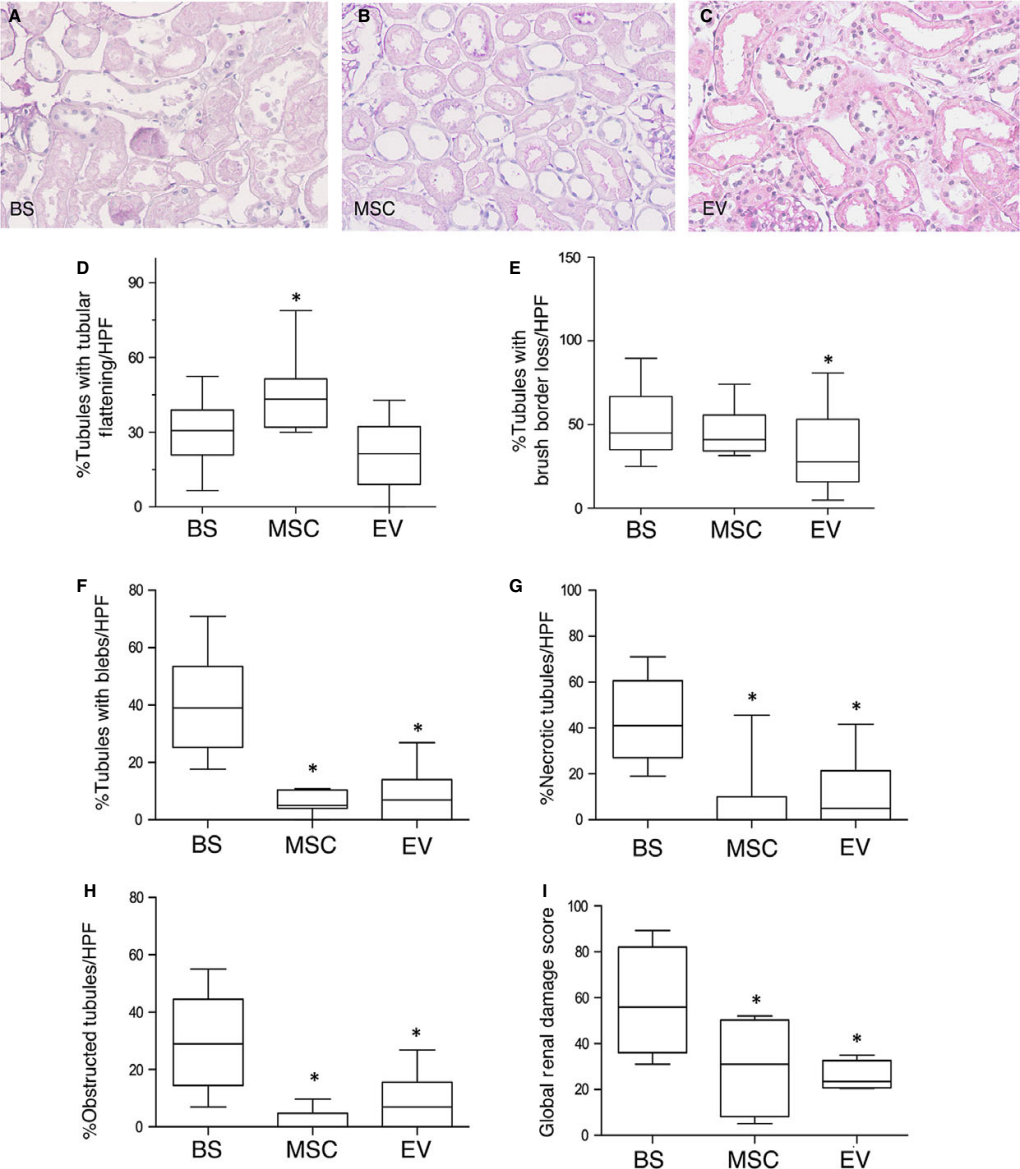
Ischaemic renal damage in BS and MSC‐/EV‐perfused kidneys. Representative renal sections of kidneys perfused after 20 min. of ischaemia either with Belzer solution (BS) (panel **A**), or Belzer solution supplemented with 3 million MSC (MSC) (panel **B**) or Belzer solution supplemented with EV derived from 3 million MSC (EV) (panel **C**). PAS staining, magnification ×200. Panels **D‐I**. Boxplots showing the distribution of renal lesions in all groups. Box: median, 25–75° percentile; whiskers, 5–95° percentile. Data are the percentage of tubules/HPF in which the lesions were observed. Panel **D**. *P < 0.05 *versus* BS and EV. Panel **E**. *P < 0.05 *versus* BS. Panel **F**. *P < 0.0001 *versus* BS. Panel **G**. *P < 0.0001 *versus* BS. Panel **H**. *P < 0.0001 *versus* BS. Panel **I**. *P < 0.0001 *versus* BS. TF: tubular epithelial cell flattening, BBL: brush border loss, BF: bleb formation, TO: tubular lumen obstruction, TN: tubular necrosis

Figure 3 shows renal lesions evaluated in the DCD model. TF (Panel A) and BBL (Panel B, blue arrow), BF (Panel B, red arrow), TN and TO (Panel C, white arrow TN and yellow arrow TO). Figure 4A–C shows representative renal sections of perfused kidneys. The prevalence of different types of lesions in BS‐, MSC‐ and EV‐perfused kidneys is reported in Figure 4D–I.

Tubular epithelial cell flattening was significantly more present in tubules from MSC‐perfused kidneys (43.2%) *versus* BS (30.7%) and EV‐perfused kidneys (21.3%) (Fig. 4D), BBL was more pronounced in MSC (40.9%) and BS (45.0%) compared to EV (27.7%) kidneys (Fig. 4E). Severe lesions were more evident in BS than in MSC and EV kidneys: in BS, 39.0% of tubules presented with blebs *versus* 5% in MSC and 7.0% in EV (Fig. 4F), TN was more evident in BS (41.0%) than in MSC (0%) and EV (5.0%) (Fig. 4G) and lumen obstruction was significantly present in BS (29.0%) and almost absent in MSC (0%) and EV (7.0%) (Fig. 4H). The global renal damage score was significantly more severe in kidneys perfused with BS (55.9%) than MSC (31.1%) and EV (23.5%) (Fig. 4I).

### Cell energy metabolism and membrane transport genes were up‐regulated in MSC‐/EV‐perfused kidneys

**Figure 5:**
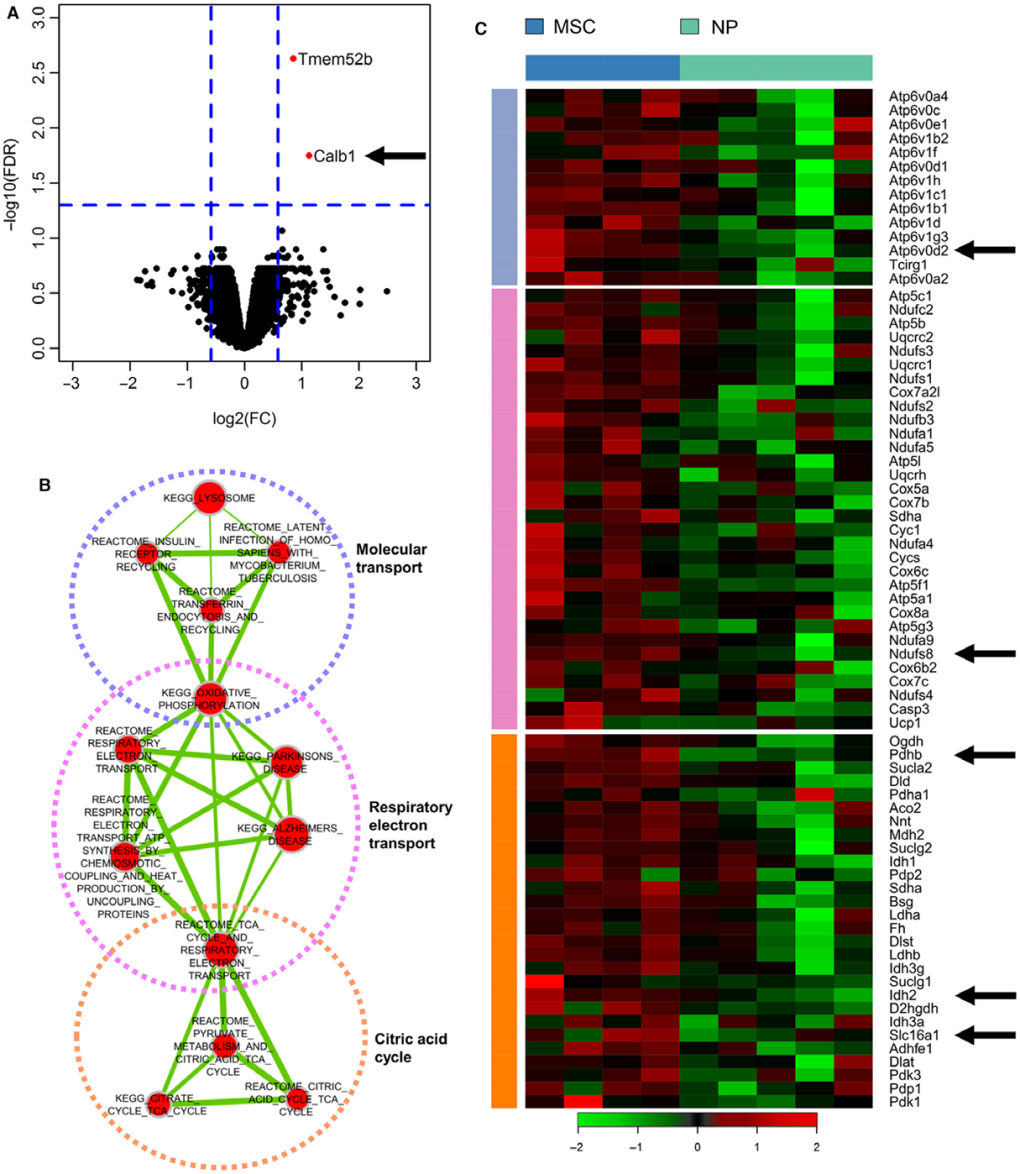
Microarray gene analysis. Gene expression profiling of kidneys perfused with MSC (MSC) compared with non‐perfused kidneys (NP). Panel **A**. Volcano plot of log_2_ fold changes *versus* –log_10_ FDR showing transcriptional differences between MSC‐perfused samples and controls. Vertical dashed lines represent the 1.5‐fold change cut‐off, and the horizontal dashed line denotes the 0.05 FDR cut‐off. Panel **B**. Enrichment map of significantly enriched gene sets at *P* < 0.001 and FDR < 0.05. Nodes represent gene sets connected by an edge when they share common genes. Node size is proportional to the number of genes in the gene set, and edge thickness is proportional to the overlap between gene sets. Red gene sets are enriched in MSC‐perfused samples, blue gene sets in controls. Highly interconnected gene sets are biologically related and were manually annotated into macro‐categories. Panel **C**. Heatmap showing the expression of core genes, identified by leading edge analysis of metabolic gene sets, in MSC‐perfused and control samples. Only genes shared by at least two gene sets were selected. Vertical bars are colour‐coded according to gene set clusters in panel **B**.

After hybridization onto the microarray, one of the MSC samples resulted as poor quality and was discarded. Therefore, the comparison was made between five NP samples and four MSC samples. Differential expression analysis between the two biological classes revealed only two genes that were significantly up‐modulated (fold change >1.5 and FDR < 0.05) in MSC‐treated kidneys: Tmem52b, encoding a transmembrane protein, and Calb1 that encodes a calcium binding protein (Fig. 5A). No significantly down‐regulated genes were found. To interpret the biology underlying and identify subtle differences commonly present in gene sets, we applied GSEA for the identification of perturbed biological processes after MSC perfusion. We identified 20 gene sets that were significantly enriched in MSC‐perfused samples; of these, 13 were related to molecular transport, respiratory electron transport, the citric acid cycle and some of them overlapped these categories (Fig. 5B) (see Fig. S1 for the complete list of significantly enriched gene sets).

We focused our attention on metabolic processes and the identification of core genes driving the enrichment of these gene sets. We performed a cutting edge analysis and selected those occurring in at least two gene sets. The expression of these genes in MSC‐perfused and NP samples is shown in Figure 5C. We selected five genes for further analyses, those with a key role in energetic metabolism and membrane transport.

From the microarray analysis, we selected three genes encoding proteins that contribute to improved mitochondrial activity, ATP synthesis and BS antioxidant effects (Idh2: isocitrate dehydrogenase 2; Ndufs8: NADH dehydrogenase Fe‐S protein 8; Pdhb: pyruvate dehydrogenase beta) and three genes encoding proteins involved in membrane transport and cellular homoeostasis preservation (Calb1: Calbindin 1; Slc16a1: monocarboxylate transporter 1; Atp6v0d2: vacuolar H+‐ATPase d2 Subunit).

### Idh2, Ndufs8 and Pdhb mRNA expression was up‐regulated in MSC/EV renal tissue, Calb1, Slc16a1 and Atp6v0d2 mRNA was up‐regulated in EV renal tissue

**Figure 6:**
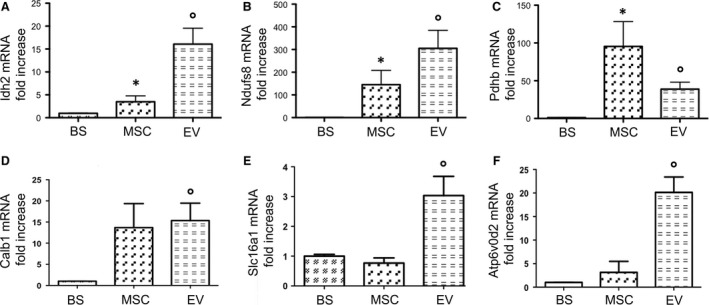
mRNA expression of cell energy metabolism and membrane transport genes validated by RT‐PCR in BS, MSC and EV groups. Columns show gene mRNA expression normalized to beta‐actin expression and converted to fold change. Data are means ± S.D. The groups are defined in Figure 4. Panel **A**. Isocitrate dehydrogenase 2 (Idh2) mRNA expression (°*P* < 0.005 *versus* BS; *P < 0.05 *versus* EV and BS). Panel **B**. NADH dehydrogenase (ubiquinone), Fe‐S protein 8 (Ndufs8) mRNA expression (°*P* < 0.005 *versus* BS; *P < 0.05 *versus* BS). Panel **C**. Pyruvate dehydrogenase beta (Pdhb) mRNA expression (*P < 0.05 *versus* BS; °*P* < 0.01 *versus* BS). Panel **D**. Calbindin1 (Calb1) mRNA expression (°*P* < 0.05 *versus* BS). Panel **E**. Monocarboxylate transporter 1 (Slc16a1) mRNA expression (°*P* < 0.05 *versus* BS and MSC). Panel **F**. Vacuolar H + ‐ATPase d2 Subunit (Atp6v0d2) mRNA expression (°*P* < 0.01 *versus* BS and MSC).

RT‐PCR of selected genes was performed in renal tissues from the BS, MSC and EV groups (*n* = 5 for each group). This analysis showed that mRNA expression of Idh2, Ndufs8 and Pdhb was significantly higher in kidneys perfused with MSC and EV than in those perfused only with BS (Fig. 6A–C).

Moreover in EV, but not in MSC‐perfused kidneys, we found the up‐regulation of Calb1, Slc16a1 and Atp6v0d2 mRNA in comparison with BS group (Fig. 6D‐F).

### Markers of ischaemic damage and glucose were lower, pyruvate was higher in MSC/EV than BS effluent fluid

**Figure 7:**
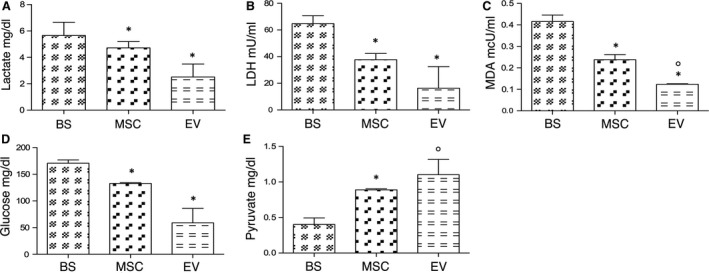
Markers of ischaemic damage and energy substrates in effluent fluid. Columns show levels of lactate (mg/dl), LDH (mU/ml), malondialdehyde (MDA) (mcU/ml), glucose (mg/dl) and pyruvate (mg/dl) in effluent fluid from each group. Data are means ± S.D. The groups are defined in Figure 4. Panel **A**. *P < 0.05 *versus* BS. Panel **B**. *P < 0.05 *versus* BS. Panel **C**. *P < 0.05 *versus* BS; °*P* < 0.01 *versus* MSC. Panel **D**. *P < 0.05 *versus* BS. Panel **E** *P < 0.05 *versus* BS; °*P* < 0.005 *versus* BS.

After 4 hrs of perfusion at 4°C with BS alone or in the presence of either MSC or EV, the effluent fluid was collected and stored at −20°C till the time of analysis. The markers of disease activity 40, LDH and lactate and of oxidative stress and lipid peroxidation 41. MDA were significantly higher in effluent fluid of BS kidneys (lactate 5.66 ± 0.99 mg/dl; LDH 64.75 ± 11.90 mU/ml; MDA 0.41 ± 0.06 mcU/ml) compared to MSC (lactate 3.12 ± 0.12 mg/dl; LDH 37.67 ± 8.1 mU/ml; MDA 0.23 ± 0.05 mcU/ml) and EV kidneys (lactate 2.51 ± 0.98 mg/dl; LDH 16.33 ± 16.23 mU/ml; MDA 0.12 ± 0.004 mcU/ml) (Fig. 7A–C). Glucose levels in effluent fluid from BS kidneys were significantly higher (170.8 ± 13.77 mg/dl) than in MSC (132.7 ± 2.80 mg/dl) and EV (59.0 ± 7.60 mg/dl) (Fig. 7D). Pyruvate levels in MSC (0.89 ± 0.02 mg/dl) and EV effluent (1.1 ± 0.04 mg/dl) were significantly higher than in BS effluent (0.41 ± 0.20 mg/dl) (Fig. 7E).

## Discussion

It is known that hypothermic perfusion with BS ameliorates the viability of grafts 1. Here, we report in a rat model of DCD kidney that pre‐conditioning with MSC, and even more with MSC‐derived EV, results in a significant reduction in renal ischaemic injury. During ischaemia, there is a switch from aerobic to anaerobic metabolism, which leads to intracellular changes: ATP levels decline, intracellular calcium and protons increase together with mitochondrial membrane permeability, ROS rise, and lysosome enzymes are released with consequent cell structure breakage 9, 10, 11, 12, 13. In addition, hypoxia inhibits glucose oxidative phosphorylation; thus, anaerobic glycolysis remains the only source of ATP production. Conversion of pyruvate into lactate quickly overloads cells with lactate and protons; hence, lactate becomes the leading actor in cellular metabolism. Proton excess disrupts phospholipid membrane integrity, while an ATP shortage deprives ion transporters of fuel causing intracellular ion accumulation and cell swelling 42, 43. All these events result in cellular architecture subversion and morphological changes 44, 45. Even though in our experimental groups, a heterogeneous pattern of lesions was observed, the global renal damage score was significantly lower in MSC/EV as compared with BS kidneys, where a higher percentage of severe lesions was observed. The damage progression in MSC‐perfused kidneys was limited to early stages, while EV were the most effective in stopping the progression of ischaemic injury.

The gene expression array analysis shed light on why MSC/EV delivery to the isolated organ provides protection for the DCD kidney. In fact, gene pathways for molecular transport, respiratory electron transport and the citric acid cycle were significantly up‐regulated in MSC compared to NP. The results obtained comparing MSC with NP kidneys were studied in BS, MSC and EV kidneys by RT‐PCR mRNA expression of six key genes, whose functions could enhance BS efficiency and improve cell homoeostasis during cold perfusion.

Overall, analyses of the biochemical products in perfused kidney effluents were in agreement with the histopathological data and supported by the final effectors of gene pathways up‐regulated in MSC/EV kidneys. In fact, in BS effluent fluid the ischaemic damage marker levels (LDH, lactate) and MDA, expression of oxidative stress and lipid peroxidation, were significantly higher than in MSC/EV effluent. Improved MSC/EV kidney viability was also justified by the effluent levels of glucose and pyruvate, important intermediates in energy metabolism. Compared with BS, effluent glucose levels were lower in MSC/EV kidneys, while pyruvate levels were higher possibly indicating an increased glucose conversion into pyruvate. On the other hand, it is known that ischaemia induces persistent pyruvate depletion 42 and pyruvate administration during ischaemia protects kidneys from injury;^14^, ^46^, ^47^ thus, we propose that the higher levels of pyruvate preserved MSC/EV kidneys through its antioxidant and anti‐inflammatory effects 42, 48, 49.

**Figure 8:**
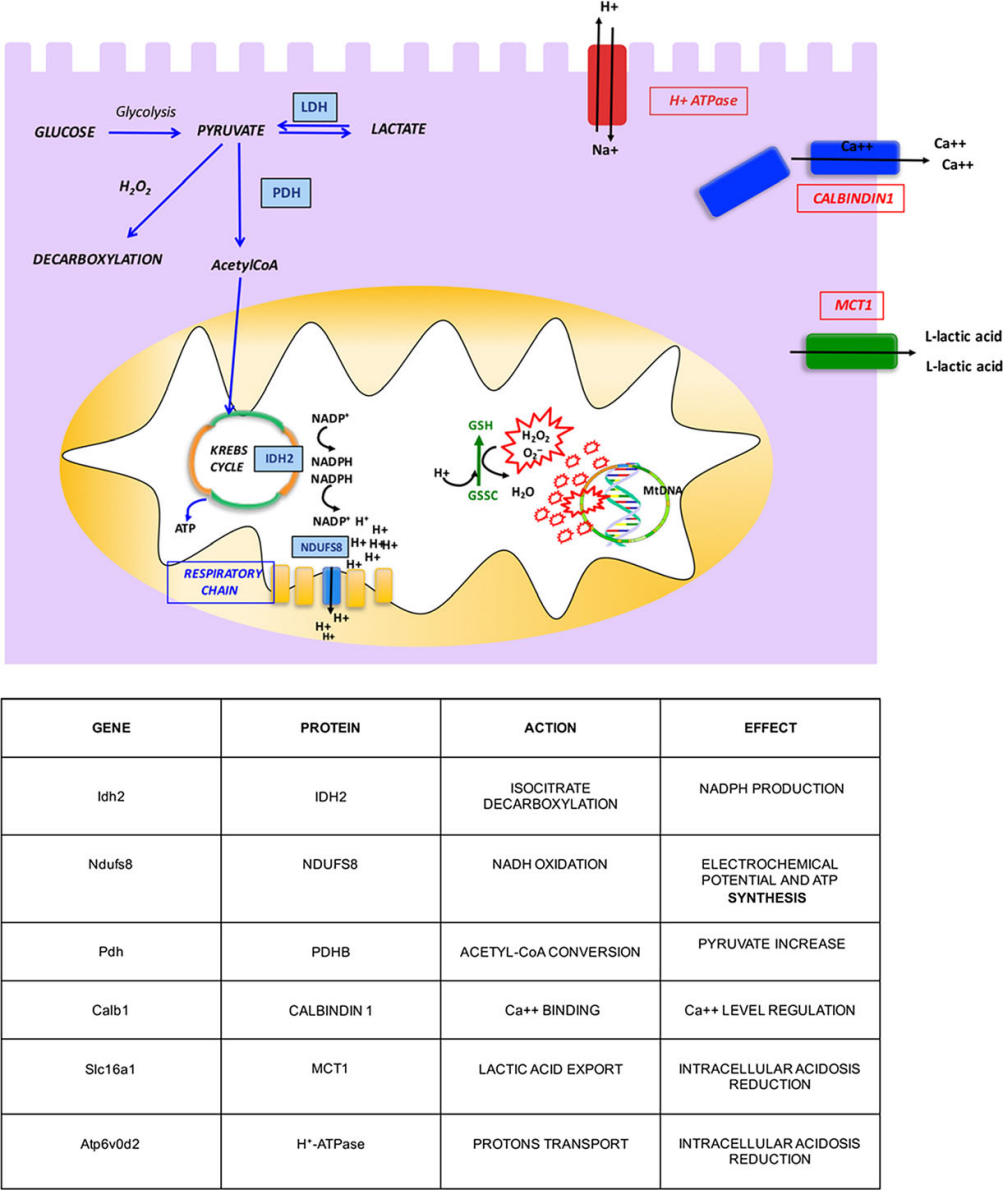
Schematic representation of cell energy metabolism pathways and ion membrane transporters up‐regulated in MSC/EV groups.

The cell energy metabolism pathways and ion membrane transporters up‐regulated in the MSC/EV groups, and their effectors are reported in Figure 8.

The Idh2 gene encodes for isocitrate dehydrogenase 2, which generates NADPH, and is indispensable for producing reduced glutathione (GSH), potent antioxidant 50, 51, 52, 53, 54. Thus, we think that Idh2 upregulation in MSC‐/EV‐perfused kidneys could provide greater NADPH availability and ensure that glutathione, supplied by BS, ameliorates its antioxidant activity. Similarly, the Ndufs8 gene, which encodes NADH‐ubiquinone oxidoreductase, induces a proton flux and an electrochemical potential across the mitochondrial membrane that drives ATP synthesis 55, 56, 57, 58. Hence, the greater ubiquinone availability, together with adenosine provided by BS, may accelerate ATP synthesis reducing energy depletion. Our hypothesis is supported by evidence from an ischaemia–reperfusion model where the administration of reduced ubiquinone improves renal damage, by enhancing electron transport, preventing ROS generation and increasing ATP production 59. The Pdhb gene, encoding pyruvate dehydrogenase beta, has a key role in ischaemic cell energy metabolism. It is known that acute kidney ischaemia decreases PDH and pyruvate levels, increases lactate levels 42 and that pyruvate administration protects from ischaemic injury 14, 46, 47, 48, 49. As Figure 8 shows, pyruvate is central to many metabolic pathways: (*i*) glycolysis, which leads to pyruvate generation; (*ii*) pyruvate decarboxylation by PDH, which leads to acetyl‐CoA formation; (*iii*) pyruvate–lactate conversion; and (*iv*) pyruvate decarboxylation during H_2_O_2_ scavenging. In MSC/EV kidneys, we observed upregulation of the Pdhb gene, that potentially should increase pyruvate to acetyl‐CoA conversion resulting in a decrease of pyruvate levels in effluent fluid. Experimentally, we observed a pyruvate rise, but this could be explained by the reduction in LDH levels and pyruvate–lactate conversion, and by the decreased pyruvate consumption by H_2_O_2_‐mediated decarboxylation (H_2_O_2_ scavenging). In fact, MDA levels were lower in treated kidneys. Calb1 encodes calbindin1, a calcium binding protein, that plays a pivotal role in intracellular calcium regulation and prevents calcium toxicity 60. Ca2^++^ overload causes oxidative stress generating ROS and induces apoptosis and cell death opening mitochondrial transition pores 10, 11, 61. Several studies have already demonstrated the protective effect of calbindin1 in experimental models of ischaemic stroke 62, retinal ischaemia 63 and cyclosporine A toxicity in tubular cells 64. Slc16a1 encodes for MCT1, an almost ubiquitous protein whose role is to facilitate L‐lactic acid transmembrane movements. During hypoxia or anoxia, MCT1 exports lactic acid accumulated during anaerobic glycolysis, reducing intracellular acidosis 43, 65, 66, 67. The protective effect of MCT1 upregulation and consequent lactate export has already been described in myocardial ischaemia–reperfusion injury 68. The Atp6v0d2 gene encodes subunit isoforms of H + ‐ATPase which transports protons across the cellular membrane; this action is required to reduce intracellular acidosis 69, 70, 71. Interestingly, the last three genes were significantly up‐regulated only in EV‐perfused kidneys in agreement with their better histopathological preservation.

We hypothesize that the major effectiveness of EV *versus* MSC may depend on the prompt availability of MSC mediators contained in EV. Furthermore, EV were released by MSC, conditioned overnight in DMEM without FCS; therefore, they were committed to an energy depletion setting 28. *In vitro* studies have shown that the EV incorporation rate was accelerated in ATP‐depleted tubular cells 28 and correlated positively with intracellular proton concentration 72. Furthermore, the release of EV from primary cultures of cytotrophoblast cells was inversely correlated with oxygen tension. 73 These findings suggest that soluble EV are more promptly available to hypoxic cells than EV delivered by MSC. Therefore, free EV would oppose the cascade of reactions sparked by ischaemia in an earlier stage and be more effective in preventing ischaemic injury. EV contribute to MSC paracrine action by delivering microRNA, mRNA, long non‐coding RNA and occasionally genomic DNA into target tissues and can bind target cells through specific receptors and transfer proteins, lipids, mRNAs and miRNAs 25, 26, 27, 28. Recently, it has been reported that MSC‐derived EV modulate miRNA in renal tubular cells and inhibit ATP depletion injury 28. In glycerol 26, cisplatin 25 and ischaemia–reperfusion model of acute kidney failure 74, the intravenous administration of EV or MSC had the same efficacy on functional and morphological recovery.

In conclusion, we have demonstrated that MSC/EV administration in addition to BS, normally used in HMP for DCD organs, blocks renal ischaemic damage at an early stage by preserving the enzymatic machinery essential for cell viability and prepares the kidney for reperfusion damage. To the best of our knowledge, this is the first report on new gene pathways by which MSC/EV may act in tissue protection/repair. Even though the *in vivo* portion of the study was limited, for example, the transplantation of treated kidneys was not performed, we strongly believe that our results should pave the way for more in depth, *in vivo* studies; in fact, the future steps of this project will be the identification of mediators involved in the better effectiveness of EV and the transplant of kidneys conditioned with MSC or EV.

## Authorship

G.M. contributed to the research design, and she was responsible for the histological studies, contributed to the analysis and interpretation of data, wrote and approved the final version of the manuscript. C.V. performed the microsurgery experiments and contributed to the collection and analysis of data. P.E.F. contributed to the research design, histological studies and collection of data. R.C. and M.S. performed the RT‐PCR analysis. P.A. performed the microsurgery experiments. C.S. was responsible for the microarray analysis. D.C.L and D.M. performed the microarray analysis. A.M.A. was responsible for MSC and EV isolation and characterization, revised and approved the final version of the manuscript. M.M. contributed to MSC and EV isolation and characterization. B.S. contributed to EV characterization. M.Ma supervised the microsurgical experiments. E.P. and L.C. approved the final version of the manuscript. D.C.A. critically revised and approved the final version of the manuscript, R.T. designed and supervised the experiments, wrote and approved the final version of the manuscript.

## Conflict of interests

The authors have no conflict of interests to declare.

## Supporting information


**Figure S1**. Heatmap reporting leading edge genes (genes that drive the significance of a gene set) for gene sets significantly altered (FDR < 0.05) between MSC and NP samples. Red: genes included in the gene set; grey: genes not included in the gene set.
