# Supplementary material for: Perfusion of isolated rat kidney with Mesenchymal Stromal Cells/Extracellular Vesicles prevents ischaemic injury
Source: J Cell Mol Med. 2017 Jun 21;21(12):3381–93. doi: 10.1111/jcmm.13249 (PMC5706569; doi:10.1111/jcmm.13249)
Supplement: Supplementary file 1 — Figure S1. Heatmap reporting leading edge genes (genes that drive the significance of a gene set) for gene sets significantly altered (FDR < 0.05) between MSC and NP samples. Red: genes included in the gene set; grey: genes not included in the gene set. [file JCMM-21-3381-s001.pdf]

SUPPLEMETARY FIGURES

Heatmap reporting leading edge genes (genes that drive the significance of a gene set) for gene sets significantly altered (FDR<0.05) between MSC and NP samples. Red: genes included in the gene set; grey: genes not included in the gene set.

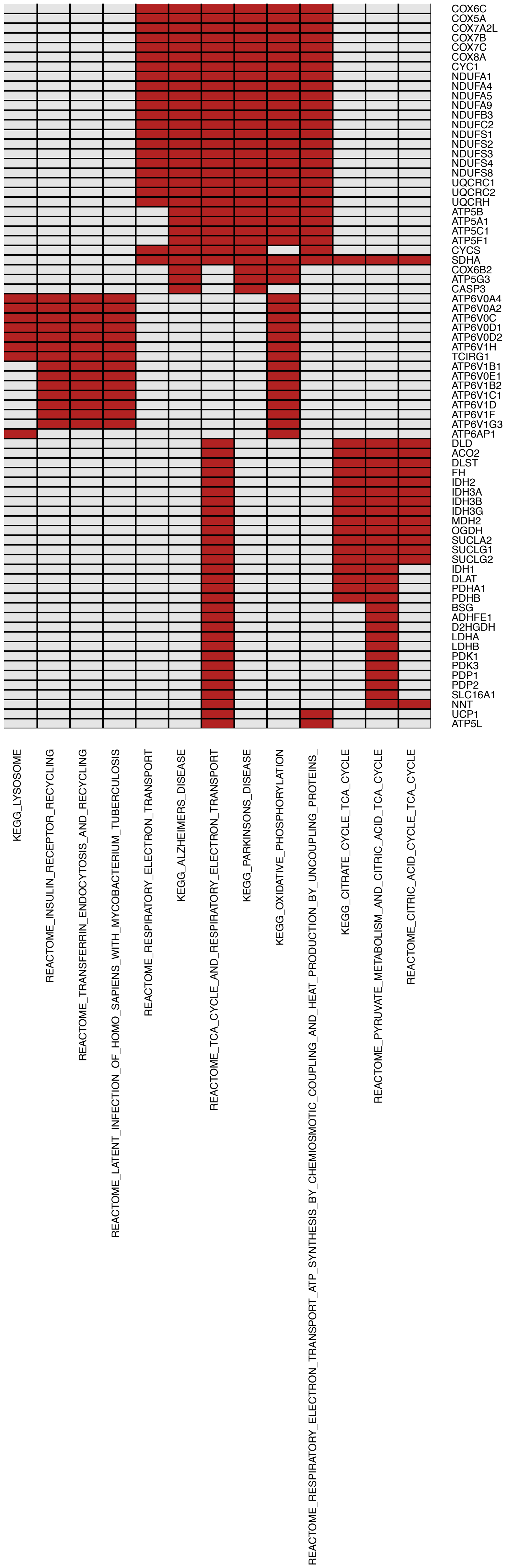
COX6C
COX5A
COX7A2L
COX7B
COX7C
COX8A
CYC1
NDUFA1
NDUFA4
NDUFA5
NDUFA9
NDUFB3
NDUFC2
NDUFS1
NDUFS2
NDUFS3
NDUFS4
NDUFS8
UQCRC1
UQCRC2
UQCRH
ATP5B
ATP5A1
ATP5C1
ATP5F1
CYCS
SDHA
COX6B2
ATP5G3
CASP3
ATP6V0A4
ATP6V0A2
ATP6V0C
ATP6V0D1
ATP6V0D2
ATP6V1H
TCIRG1
ATP6V1B1
ATP6V0E1
ATP6V1B2
ATP6V1C1
ATP6V1D
ATP6V1F
ATP6V1G3
ATP6AP1
DLD
ACO2
DLST
FH
IDH2
IDH3A
IDH3B
IDH3G
MDH2
OGDH
SUCLA2
SUCLG1
SUCLG2
IDH1
DLAT
PDHA1
PDHB
BSG
ADHFE1
D2HGDH
LDHA
LDHB
PDK1
PDK3
PDP1
PDP2
SLC16A1
NNT
UCP1
ATP5L
KEGG_LYSOSOME
REACTOME_INSULIN_RECEPTOR_RECYCLING
REACTOME_TRANSFERRIN_ENDOCYTOSIS_AND_RECYCLING
REACTOME_LATENT_INFECTION_OF_HOMO_SAPIENS_WITH_MYCOBACTERIUM_TUBERCULOSIS
REACTOME_RESPIRATORY_ELECTRON_TRANSPORT
KEGG_ALZHEIMERS_DISEASE
REACTOME_TCA_CYCLE_AND_RESPIRATORY_ELECTRON_TRANSPORT
KEGG_PARKINSONS_DISEASE
KEGG_OXIDATIVE_PHOSPHORYLATION
REACTOME_RESPIRATORY_ELECTRON_TRANSPORT_ATP_SYNTHESIS_BY_CHEMIOSMOTIC_COUPLING_AND_HEAT_PRODUCTION_BY_UNCOUPLING_PROTEINS_
KEGG_CITRATE_CYCLE_TCA_CYCLE
REACTOME_PYRUVATE_METABOLISM_AND_CITRIC_ACID_TCA_CYCLE
REACTOME_CITRIC_ACID_CYCLE_TCA_CYCLE
